# Microvascular Pathology and Morphometrics of Sporadic and Hereditary Small Vessel Diseases of the Brain

**DOI:** 10.1111/bpa.12177

**Published:** 2014-10-16

**Authors:** Lucinda J.L. Craggs, Yumi Yamamoto, Vincent Deramecourt, Raj N. Kalaria

**Affiliations:** ^1^ Institute for Ageing and Health Newcastle University Newcastle upon Tyne UK; ^2^ Department of Regenerative Medicine and Tissue Engineering National Cerebral and Cardiovascular Center National Cerebral and Cardiovascular Center Research Institute Osaka Japan; ^3^ Department of Histology University Lille Nord de France Lille France

**Keywords:** arteriopathy, CADASIL, cognitive impairment, leukoencephalopathy, molecular genetics, small vessel disease, stroke, white matter

## Abstract

Small vessel diseases (SVDs) of the brain are likely to become increasingly common in tandem with the rise in the aging population. In recent years, neuroimaging and pathological studies have informed on the pathogenesis of sporadic SVD and several single gene (monogenic) disorders predisposing to subcortical strokes and diffuse white matter disease. However, one of the limitations toward studying SVD lies in the lack of consistent assessment criteria and lesion burden for both clinical and pathological measures. Arteriolosclerosis and diffuse white matter changes are the hallmark features of both sporadic and hereditary SVDs. The pathogenesis of the arteriopathy is the key to understanding the differential progression of disease in various SVDs. Remarkably, quantification of microvascular abnormalities in sporadic and hereditary SVDs has shown that qualitatively the processes involved in arteriolar degeneration are largely similar in sporadic SVD compared with hereditary disorders such as cerebral autosomal arteriopathy with subcortical infarcts and leukoencephalopathy (CADASIL). Important significant regional differences in lesion location within the brain may enable one to distinguish SVDs, where frontal lobe involvement appears consistently with almost every SVD, but others bear specific pathologies in other lobes, such as the temporal pole in CADASIL and the pons in pontine autosomal dominant microangiopathy and leukoencephalopathy or PADMAL. Additionally, degenerative changes in the vascular smooth muscle cells, the cerebral endothelium and the basal lamina are often rapid and more aggressive in genetic disorders. Further quantification of other microvascular elements and even neuronal cells is needed to fully characterize SVD pathogenesis and to differentiate the usefulness of vascular interventions and treatments on the resulting pathology.

## Introduction

Small vessel disease (SVD) of the brain is common in community‐dwelling elderly subjects 81, 93, 96. It is now widely accepted that subcortical ischemic vascular dementia (VaD) results from SVD 90, 94. The pathogenesis of SVD is still relatively poorly understood and therapeutic strategies are limited. Neuroradiologically, SVD is recognized by focal ischemic lesions or lacunes in the subcortical structures and by diffuse white matter hyperintensities on T2‐weighted magnetic resonance imaging (MRI) described as leukoaraiosis 90, 97. Patients with SVD exhibit motor and executive slowing, forgetfulness and dysarthria. A short‐stepped gait is also common and can mimic that of Parkinsonism. These may be caused by disruption of pathways running from the prefrontal cortex to the basal ganglia and of thalamocortical pathways 94. “Pure” subcortical VaD with a slowly progressive course may mimic Alzheimer's disease (AD) but in the general absence of the characteristic brain neurofibrillary burden. The main vascular pathological features involve sclerotic changes in intracranial arteries and arterioles, whereas parenchymal lesions in the subcortical structures largely involve lacunar infarcts, microinfarcts, increased perivascular spacing and deep white matter (WM) attenuation (Table 1). However, small infarcts or microinfarcts and tissue thinning may also occur in the cortex. Highly specific categories of subcortical VaD may be due to infarctions located in the thalamus with relatively little involvement of other brain structures 15.

**Table 1:** Sporadic vs. hereditary small vessel diseases of the brain. Abbreviations: CARASIL = cerebral autosomal recessive arteriopathy with subcortical infarcts and leukoencephalopathy; GOM = granular osmiophilic material; HSA = hereditary systemic angiopathy; HERNS = hereditary endotheliopathy with retinopathy, nephropathy and stroke; PADMAL = pontine autosomal dominant microangiopathy and leukoencephalopathy; PAS = periodic acid–Schiff; RVCL = retinal vasculopathy with cerebral leukodystrophies; WM = white matter

Disorder/ Inheritance pattern	Onset age (years)^b^	Duration of disease (years)	Key clinical features^a^	Ophthalmological findings	Neuroimaging findings	Pathological features	Genetic trait(s)	References
Sporadic SVD	65–80	10–12	Primary deficits in executive functioning, alongside motor hemiparesis, bulbar signs and dysarthria, gait disorder, depression and emotional lability	Narrower central retinal arterioles and arteriovenous nicking predictive of lacunar stroke	WM lesions, lacunes and microbleeds	Vessel arteriosclerosis, liphyalinosis, arteriolosclerosis of subcortical vessels. Loss of vascular smooth muscle cells. Lacunar infarcts, microinfarcts, microbleeds	NOTCH3 polymorphisms, APOE, renin–angiotensin system (RAS)	Schmidt *et al* 97, Kalaria and Erkinjutti 61 Jellinger 56
CADASIL	6–48, average age 30	Average 25	Migraine with aura, transient ischemic attacks and ischemic strokes, mood disturbances (depression and apathy), eventual cognitive impairment (beginning with decreased executive function and processing speed) with motor impairment, gait disturbances, and pseudobulbar palsy	Arteriolar sheathing, arteriolar narrowing and arteriovenous nicking in a study of 10 cases	Ischemic infarcts, lacunes and diffuse leukoencephalopathy, located within the periventricular WM, basal ganglia, thalamus, internal capsule and the pons	Cerebral vessels are consistently narrowed by intimal thickening, degeneration of smooth muscle cells in vessel wall, deposition of the GOM	*NOTCH3*	Chabriat *et al* 17; Kalimo *et al* 64; Haritoglou *et al* 47
Hereditary multi‐infarct dementia of the Swedish type/ Autosomal dominant	29–38	9–13	Stroke episodes with pyramidal, bulbar and cerebellar symptoms Progressive cognitive dysfunction	None reported	Diffuse WM lesions, lacunar strokes and atrophy	Subcortical lacunes, arteriopathy, splitting of elastic lamina, no presence of GOM	Not linked to CADASIL locus	Low *et al* 75; Sourander and Walinder 105; Zhang *et al* 128
PADMAL Subcortical angiopathic encephalopathy/ Autosomal dominant	12–50	4–33	Recurrent strokes, gait disturbance, dysarthria, sensorimotor deficits and progressive dementia	None reported except 1 case with contusional hemianopsia	Large confluent areas of WM changes, necrosis in brainstem, particularly pons, basal ganglia and WM	Lacunar infarcts, arteriopathy, demyelination, degeneration of pyramidal tracks and corpus callosum. Microvascular changes, no PAS + ve deposits or GOM	Not linked to CADASIL or RVCL locus	Colmant 18; Hagel *et al* 45; Ding *et al* 25
CARASIL (Maeda syndrome)/ Autosomal recessive	20–30	5–20	Recurrent small strokes, lumbar intervertebral disc herniations, kyphosis, ossification of intraspinal ligaments, osseous deformities, alopecia. Progressive dementia	Optic neuritis and retinal vascular changes in 1 case	Diffuse WM lesions and small infarcts in basal ganglia (degenerative changes in lumbar and knee joints)	Arteriosclerotic changes, WM changes. No GOM deposition	*HTRA1* (1 patient with *NOTCH3* mutation, p.Cys174 Phe)	Yanagawa *et al* 124; Hara *et al* 46
						Hyaline degeneration and thickening and splitting of internal lamina		
RVCL/ Autosomal dominant	30–50	5–10	Strokes, pseudotumors, seizures, motor and sensory deficits, headaches, renal disease	Retinal microvessel changes, macular involvement, visual loss	Diffuse deep WM changes and lacunar strokes, edema	Arteriopathy, multiple lacunas, multilamination of basement membrane in capillaries. No signs of vasculitis	*TREX1*	Jen *et al* 57; Ophoff *et al* 88
HERNS (Chinese descent)								
CRV (cerebroretinal vasculopathy)	30–50	5–10	Strokes, migraines, pseudotumors, renal disease (some), dementia	Retinal capillary obliteration progressive visual loss	Diffuse WM changes edema, lacunar infarcts neurovascular changes	Not determined	*TREX1*	Grand *et al* 42; Ophoff *et al* 88
HVR (hereditary vascular retinopathy)	30–50	7–10	Strokes, Raynaud phenomenon, migraine like symptoms, visual loss	Microaneurysms, telangiectatic capillaries (aromi macula) in eye	Diffuse WM changes upon MRI unclear	Not determined	*TREX1*	Ophoff *et al* 88; Terwindt *et al* 108
HSA	40–50	∼10	Strokes, visual impairment, migraine like headaches, skin rashes, seizures, motor paresis, cognitive decline	Ischemic retinopathy, optic disc atrophy, capillary aneurysms	Multiple cerebral calcifications and tumor‐like subcortical WM lesions	Severe arteriopathy, coagulative necrosis, perivascular inflammation, edema, astrocytic gliosis	Absence of *NOTCH3* mutations.	Winkler *et al* 120
COL4‐related disorder (stroke syndrome); Autosomal dominant	14–49	>8	Infantile hemiparesis, migraines with/without aura, intracerebral hemorrhages, seizures, Raynaud phenomenon, dementia	Retinal arteriolar tortuosity, retinal haemorrhage, abnormal iris vasculature (large tortuous vessels), vascularization of cornea, optic nerve hypoplasia	Diffuse WM changes and dilated perivascular spaces, subcortical infarcts, microbleeds. Some cases have porencephaly cavities appearing as subcortical periventricular cysts.	Mice with *COL4A1* and *COL4A2* mutations exhibit severe hemorrhages and cortical defects (molecular layer heterotopia), WM defects	*COL4A1, COL4A2*	Gould *et al* 41; Vahedi *et al* 110; Volonghi *et al* 116; Kuo *et al* 68
Hereditary small vessel disease of the brain (SVDB)/ Autosomal dominant	36–52	>5	Hemiplegia, motor and some sensory deficits, memory impairment	None reported	Diffuse WM changes, cerebral deep infarcts, degeneration of pyramidal tract, multiple microbleeds	Not determined	Not linked to CADASIL locus	Verreault *et al* 113
Hereditary diffuse leukoencephalopathy with axonal spheroids (HLDS), or familial pigmentary orthochromatic leukodystrophy (POLD)	8–78 (average age 39)	9–10	Depression, anxiety, behavioral changes, and cognitive disturbance. Spastic paresis, parkinsonism, ataxia, epilepsy	None reported	Diffuse leukoencephalopathy with lacunes.	Widespread loss of myelinated fibers with neuroaxonal spheroids in WM. Spheroids are hallmark of HDLS and lipopigment deposits a hallmark feature in POLD. No conspicuous change in the cerebral cortex including vascular structures	*CSF1R*	Hoffman *et al* 52; Kinoshita *et al* 65

a Several disorders prominently characterized by leukoencephalopathy and cognitive impairment have been described in isolated families [Hirabayashi *et al* 50; Kalimo and Kalaria 63; Winkler *et al* 120 ].

b Age of onset signifies when first cerebrovascular event or gait disturbance due to spasticity was recorded.

In recent years, much knowledge has come forth from the study of several monogenic disorders, which model sporadic SVD. Many of the characteristic clinical and pathological features of these and other rarer disorders bear considerable similarities to sporadic SVD (Table 1). In particular, the pathological changes include progressive arteriopathy, subcortical strokes and WM disease. Hereditary SVDs (hSVD) have enormous implications for understanding of the pathology and mechanisms in non‐cerebral amyloid angiopathy (CAA)‐related sporadic SVD. Hereditary SVDs are caused by mutations in different genes involving structural or signaling components of vascular cells 121. Some of these include cerebral autosomal dominant arteriopathy with subcortical infarcts and leukoencephalopathy (CADASIL), cerebral autosomal recessive arteriopathy with subcortical infarcts and leukoencephalopathy (CARASIL), retinal vasculopathy with cerebral leukodystrophies (RVCL), and collagen type IV, alpha 1 (COL4A1) and alpha 2 (COL4A2)‐related disorders. Subcortical strokes lead to insidious deterioration with most subjects becoming demented in older age. Sporadic SVD characterized by WM changes on MRI has also been described to be associated with *NOTCH3* gene polymorphisms 98 and exhibits widely variable phenotypes.

SVD remains a heterogeneous disease, and therefore, one of the greatest challenges toward studying SVD and relating this to dementia lies in the lack of consistent assessment tools for both clinical and pathological measures. For example, most clinical assessments of cognition in demented cohorts tend to concentrate on memory focused cognitive assessments used in memory clinics with a tendency to focus on AD‐based dementia. Ideally, there also needs to be more detailed assessment of vascular disease‐related clinical symptoms such as impaired gait, falls, depression and incontinence 30. In addition to clinical studies, there needs to be consistent recognition of the burden of brain vascular pathology in subjects with SVD in order to relate to each patient's clinical symptoms. Achieving this would better equip us to differentiate the effects of vascular interventions for prevention of vascular cognitive impairment (VCI) and VaD. There have been various attempts by neuropathologists to generate vascular scoring tools, with the aim being one consensus set of criteria that can be used across multiple studies, and ultimately align and strengthen the datasets available for clinicopathogical studies 44, 62. As a matter of convenience, the tools tend to be semiquantitative and highly subjective, yet there is no standardized or widely accepted quantitative method for assessing vascular pathology.

This review focuses on highlighting morphological differences in age‐related sporadic and various hSVDs, other than those caused by CAA, with the intent of identifying and quantifying key features that inform about the pathogenesis of the arteriopathy and the parenchymal changes. In addition, we summarize the available methods to assess microvascular pathology and discuss some advantages of gathering quantitative data for assessment of the burden of vascular pathologies that needs attention.

## Cerebral SVD

Small vessel changes involve hyalinization of vessels, expansion of the perivascular space and pallor of adjacent perivascular myelin, with associated astrocytic gliosis 60. The smaller vessels of the brain including intracerebral end‐arteries and arterioles undergo progressive age‐related changes 69. The arteriolar changes may range from wall thickening by hyalinosis, reduction or increment of the intima to severe arteriolosclerosis and fibrinoid necrosis. The careful use of the Periodic acid–Schiff or PAS stain enables detection of any accumulation of granular material containing glycoproteins or glycolipids within the vessel walls. Uncomplicated hyalinosis is characterized by almost complete degeneration of vascular smooth muscle cells (VSMCs) (becomes acellular) with concentric accumulation of extracellular matrix components such as the collagens and fibroblasts.

**Figure 1:**
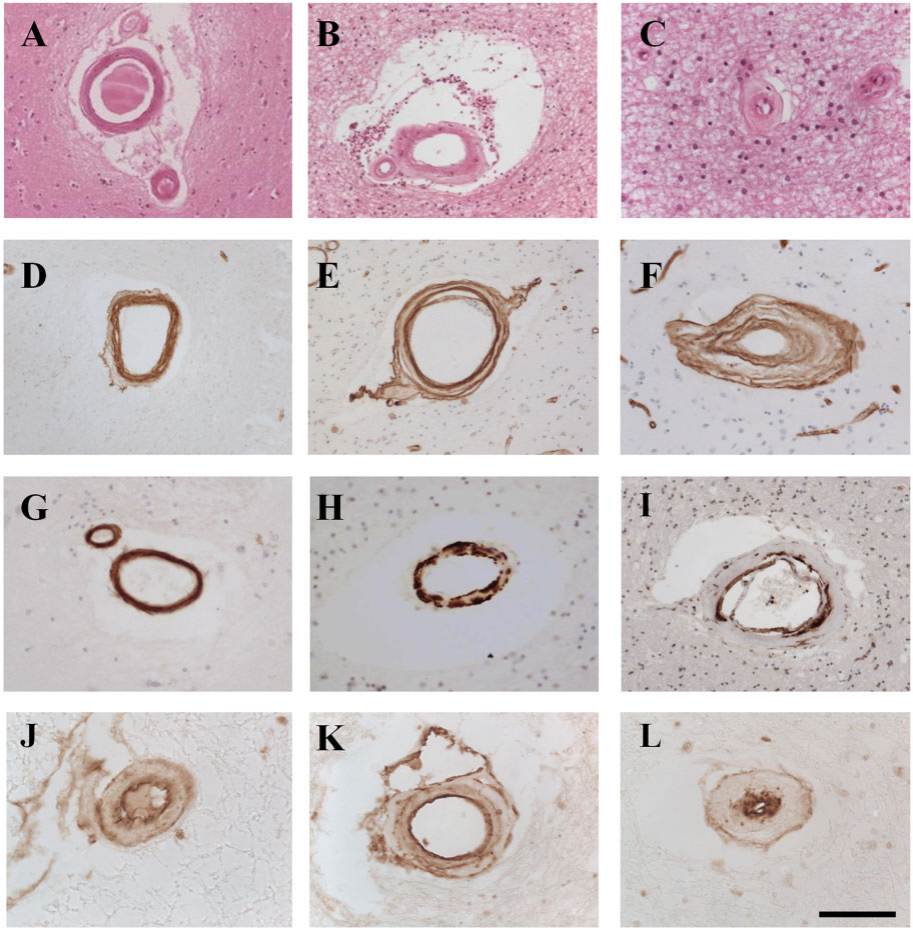
Pathological features in small vessel disease (SVD) and cerebral autosomal arteriopathy with subcortical infarcts and leukoencephalopathy (CADASIL). Panels A–C, H and E: D–F are COL4; G–I are smooth muscle alpha actin; J–L are platelet‐derived growth factor receptor‐β. A, D and G are young cognitively normal control, G is old cognitively normal control; B–K are sporadic SVD; C and L are CADASIL. Panels A–C illustrate different levels of sclerotic index in control and disease cases. A is an arteriole from cognitively control case gray matter in basal ganglia with an external diameter of 145 µm and a SI of 0.28 within the healthy range. B is an arteriole from frontal white matter (WM) in CADASIL case with an external diameter of 140 µm with an sclerotic index (SI) of 0.48 within disease state. C is an arteriole from frontal WM in CADASIL case with an external diameter of 44 µm with severe sclerosis of vessel wall with a SI of 0.77. D–F. Increased COL4 deposition observed in sporadic SVD (E) and CADASIL (F). G–I. Loss of vascular smooth muscle cells in SVD (H) and CADASIL (I) compared with cognitively normal control (G). J–L. Platelet‐derived growth factor receptor‐β staining can be observed in small pre‐capillary arterioles, which undergo hyalinosis in SVD (K) and CADASIL (L). Magnification bar = 50 µm in J and K; 70 µm in C and L; 150 µm in A, B, D, E, F G and H. 100 µm in I.

**Figure 2:**
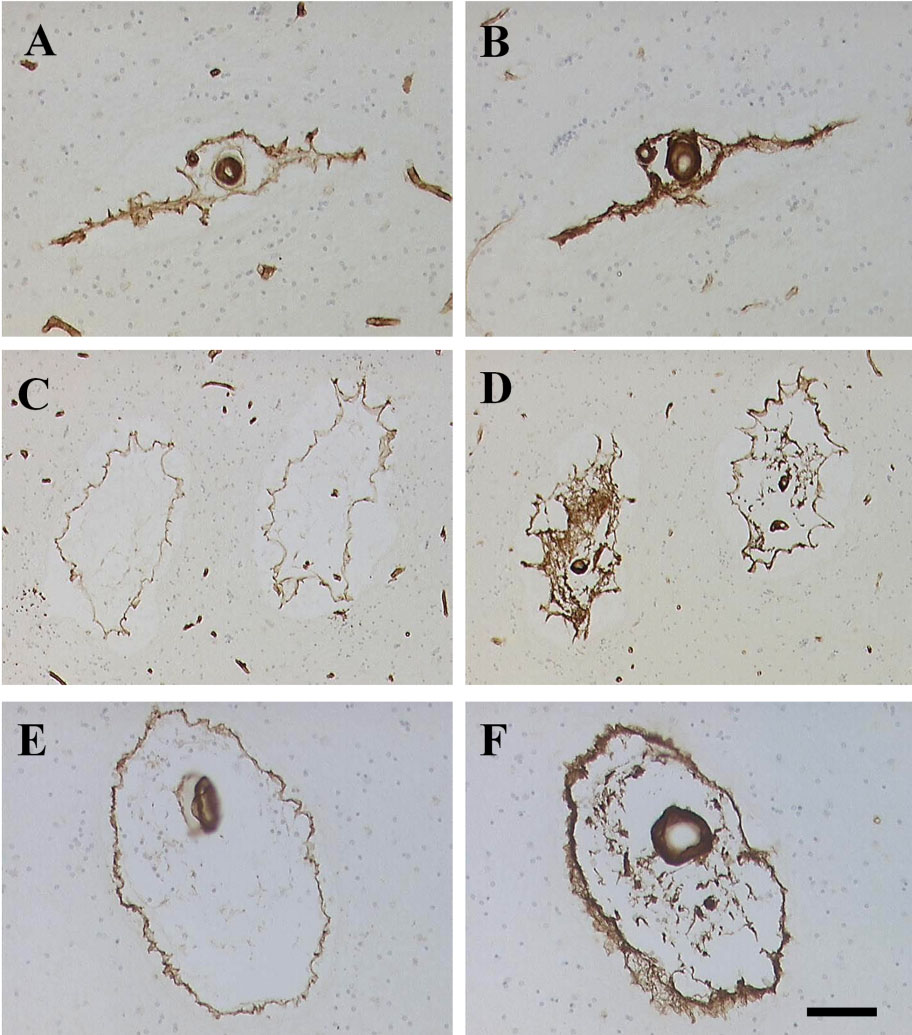
Differential arteriopathic changes detected with types of COL in cerebral autosomal arteriopathy with subcortical infarcts and leukoencephalopathy (CADASIL). Panels A, C, E show COL4 immunoreactivity in arterioles of various sizes. Panels B, D and F show COL3. The differential mobilization of COL4 and COL3 can be readily seen to determine how perivascular spaces (PVS) are caused. Note the lack of COL3 reactivity in capillaries (B and D compared with A and C). Magnification bar = 130 µm A–D; 70 µm in E and F.

Qualitatively, microvascular changes or their sequences do not appear to be necessarily different between sporadic SVD and the hSVDs. In CADASIL and CARASIL, this process is much more aggressive and intense 19 with many vessels ultimately developing a double‐barrel or wall splitting appearance, particularly in the most severe cases (Figures 1 and 2). For example, in CADASIL, medullary arteries of the frontal lobe may exhibit complete loss of medial smooth muscle cells over their entire length and severe adventitial fibrosis extending into the WM 86. Although complete occlusion is not evident, the long penetrating arterioles and their branches supplying subcortical structures are stenosed and their walls are thickened by fibrosis, conforming to increased infarcts and primary ischemic damage in the WM 79. Arteriolosclerotic changes promote loss of elasticity to dilate and constrict in response to variations of systemic blood pressure or autoregulation, which, in turn, causes fluctuations in blood flow response or hemodynamic changes to alter tissue perfusion. Depending on the size of the microvessels, perfusion changes result in lacunar infarcts (cystic lesions generally <1 cm) and microinfarcts. The deep cerebral structures and WM would be most vulnerable because the vessels are end‐arteries almost devoid of anastomoses. However, certain intrinsic arteriolar systems may be differentially affected. A recent three‐dimensional time‐of‐flight MR angiographic on 7T showed that there were no differences in luminal diameters of the lenticulostriate arteries between patients with CADASIL and control subjects. The lenticulostriate artery lumina were also not associated with lacunar infarct load in the basal ganglia area or with basal ganglia hyperintensities. On the contrary, a pathological study reported that arteriolar lumina in the lenticular nuclei were not only larger than in the WM but they were also larger than in cortical gray matter, which seldom develops infarcts 78.

**Figure 3:**
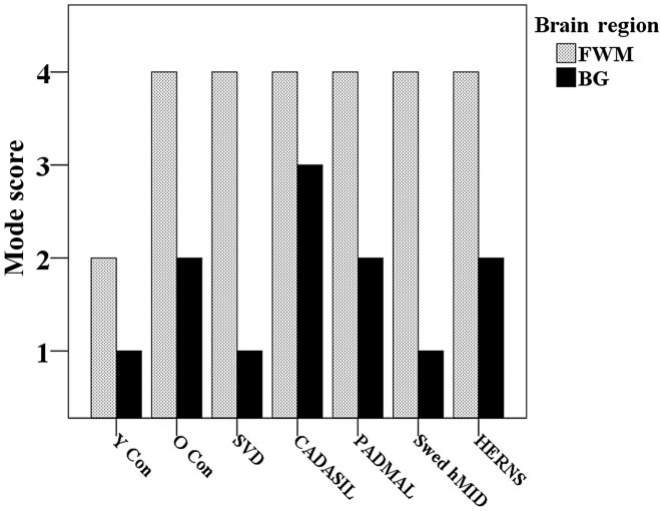
Pathological substrates of sporadic vs. hereditary small vessel diseases of the brain. Figure 1 shows the semiquantitative assessment of vascular pathology in SVDs assessed in frontal lobe (FWM) or basal ganglia (BG). Mode scores for each group were derived from a semiquantitative scale of assessment of vascular pathology giving a summary frontal score out of 6, and basal ganglia score of out of 4 [ref 24]. Abbreviations: Y Con = young controls; O Con = old controls; SVD = small vessel disease; CADASIL = cerebral autosomal dominant arteriopathy with subcortical infarcts and leucoencephalopathy; PADMAL = pontine autosomal dominant microangiopathy and leucoencephalopathy; Swed hMID = Swedish hereditary multi‐infarct dementia; HERNS = hereditary endotheliopathy with retinopathy, nephropathy and stroke.

In the early stages, small vessel changes likely lead to changes in the properties of the blood–brain barrier (BBB), with chronic leakage of fluid and macromolecules resulting in tissue edema 51, 119. Microvascular disease may also be associated with degrees of inflammation, including the presence of lymphocytes or macrophages centered on blood vessels (and not necessarily a function of brain ischemia). In the older SVD cases, there may be evidence of remote microhemorrhage in the form of perivascular hemosiderin 24. Unlike microvascular determinants (Figure 3), quantitative data on neuronal, glial or biochemical changes have largely not been fully explored across the SVDs.

## Vascular Cell Components in SVD

Various cells within the neurovascular unit, including astrocytes, VSMC, endothelial cells and pericytes, play a role in tissue perfusion and hemodynamic responses. Even subtle abnormalities in these cellular elements would accumulate to affect control of constriction and dilation as well as delivery of oxygen, glucose and nutrients to neuronal tissue.

### Mural cells

VSMCs within arteriolar walls and pre‐capillary arterioles serve as contractile elements and control blood flow responses in times of increased parenchymal demand 6, 127. Progressive pathological changes in VSMCs were described in both sporadic SVD 19 and hereditary SVDs 122. The degenerative process in CADASIL appears most aggressive almost irrespective of genotype. Loss of arterial VSMCs is followed by fibrosis of the tunica media in small‐ and medium‐sized penetrating arteries 64; arteriosclerotic changes are concomitant with stenosis, especially at the arteriolar level, through intimal thickening and wall expansion with extracellular matrix components such as collagens, laminin and fibronectin 75, 79, and compounded by altered protein–carbohydrate interactions 12, 16, 33. It is not clear whether failure in NOTCH3 signaling is also responsible for the gradual degeneration of VSMC in sporadic cases of Binswanger‐type or in hypertensive disease 86, 107. Quantitative VSMC numbers per vessel segment length in SVDs have not been fully explored, but a study in CADASIL has suggested that VSMCs undergo apoptosis akin to neurons in the neocortex 114.

### Pericytes

Pericytes juxtaposed to cerebral microvessels, most prominently wrapped around capillaries have received recent attention 22, 74. They interact with other cells within the neurovascular unit by direct contact or cell signaling mechanisms to regulate microcirculatory functions. Pericytes also likely contribute to pathogenic mechanisms in the smallest arterioles and capillaries. For example, in CADASIL, we and others 28 have observed significant abundance of granular osmiophilic material (GOM) deposits positive for NOTCH3 extracellular domain (N3ECD) around capillaries within CADASIL brains 122. Platelet‐derived growth factor receptor‐β positive pericyte‐like cells are increasingly evident in sclerosed arterioles in both CADASIL and sporadic SVD (Figure 1 and L.J.L. Craggs and R.N. Kalaria, unpub. data).

### Cerebral endothelium

Endothelial cell abnormalities and BBB dysfunction may further contribute to WM damage. Endothelial changes have been previously described in SVDs with particular reference to “blebbing,” change in volume of the cytoplasm and the presence of compact bundles of microfilaments within the cytoplasm of endothelial cells in CADASIL 75, 95, 121. Neuroimaging investigations tracking signal enhancement after gadolinium suggest that breakdown of the BBB (see previous discussion) occurs in areas of leukoaraiosis and may mediate subsequent cellular changes 117, 118. These changes are associated with chronic prothrombotic endothelial dysfunction in cerebral SVD 48 also involving the WM 11. There appears also to be a cerebral response to the SVD, both sporadic and CADASIL, by increasing endothelial thrombomodulin 38.

BBB disruption 1 can cause osmotic demyelination and result in increased permeability of the vessel wall and mobilization of inflammatory factors, such as macrophages, lymphocytes and complement, which also causes myelin damage 5. However, the projected meager perfusion due to capillary loss or abnormalities occurring prior to leukoaraiosis corroborates the finding of a chronic hypoxic state in the deep WM 32, which also releases certain growth‐promoting factors 101. It is not clear if microvascular length density increases in SVD in the most vulnerable subcortical gray or WM regions as it does in post‐stroke dementia (PSD), which suggests either an increase in angiogenesis or the formation of newer microvessel loops in response to cerebral hypoperfusion 14. However, it is likely that microvascular diameters are decreased in SVD as found in VaD, suggesting increased vasoconstriction.

### Vascular basement membrane

The basement membrane of cerebral vessels comprises several proteins, of which laminins, collagen (COL), nidogens and perlecans are the major constituents 49. The collagens are the most often investigated in the context of pathological changes in basement membranes. They are increased in hypertensive disease and associated with SVD pathology, which often reveals concentrically thickened vessel walls or hyaline arteriolosclerosis 37, and ultimately decreases the lumen diameter, impinging auto‐vasoreactivity of the vessels described as an “Earthen pipe state” 86. We evaluated COL4 staining in our cohort of hereditary and sporadic SVDs and found region‐specific differences in COL4 staining in sporadic SVD, PADMAL and Swedish hMID, where we observed increased COL4 in the frontal lobe of sporadic SVD cases, whereas the Swedish hMID cases showed increased COL4 within the basal ganglia 122. Comparisons of the different types of collagens, for example, COL3 and COL4, show the progressive changes that occur in the mobilization and restructuring of collagen‐containing vascular components (Figure 2). The pathophysiological significance of basement membrane changes is also likely to impact on clearance of fluids, solutes and toxins along the perivascular drainage route 9, 49. Enhanced COL1 and COL3 deposition has been observed in concentric rings around veins, too, termed as periventricular venous collagenosis 11, 80.

The type 4 collagens, encoded by multiple genes of which *COL4A1* and *COL4A2* are highly conserved, have gained particular importance in basement membrane changes in SVDs but also impact on large vessel disease 68. Genetic variation within both the *COL4A1* and the *COL4A2* genes have been linked to cerebral SVDs, which usually occur with systemic vascular abnormalities, causing a wide range of clinical phenotypes (Table 1). Thus, COL4A1‐related disorders show a range of phenotypes with overlapping features, including autosomal dominant type 1 porencephaly 40, brain small vessel disease with hemorrhage 41, brain small vessel disease with Axenfeld‐Rieger anomaly 99, and hereditary angiopathy with nephropathy, aneurysms and muscle cramps (HANAC) syndrome 92, 109. The consequence of altered collagen IV proteins within vessel walls is clearly catastrophic; infantile intracerebral hemorrhage and hemiparesis are common with *COL4*‐affected families, and studies in genetically modified mice expressing mutant *COL4A1* have reported 50% mortality of pups at birth, a problem that was overcome by surgical delivery 41. In adulthood, subjects with *COL4* mutations commonly have aneurysms within the carotid and cerebral arteries. Segments of the most commonly affected arteries include the intracranial carotid artery, middle cerebral artery and, least frequently, the basilar artery 71, illustrating the vital role collagens play in vascular wall structure and support.

## Parenchymal Lesions in SVD

### Lacunar infarcts

Lacunar infarcts, occurring as complete or cavitating lesions frequently in both subcortical gray and WM, are the hallmark parenchymal lesions for SVD. Neuroimaging studies suggest that lacunes occur in greater frequency in CADASIL 90 and possibly other disorders such as CARASIL. They increase with age and are associated with cognitive impairment 73, 115, but are indistinguishable between sporadic or hereditary SVD disorders. Lacunar infarcts are frequently multiple and bilateral, and often coexist with other vascular lesions, for example, large infarcts or diffuse WM damage. Interestingly, the majority of incident lacunes develop proximal to WM hyperintensities along the course of perforating vessels supplying the respective brain region. Lesion prevalence maps in different stages of disease show that lesions spread toward the subcortical regions in both sporadic SVD and CADASIL. Whether single or multiple, they may be asymptomatic, depending on their location and the loss of volume of normal brain tissue. Lacunar volumes may vary widely and do not appear to be related to age or associated cerebral lesions such as WM hyperintensities in CADASIL. However, volumes of lacunar lesions rather than that of WM hyperintensities affect cortical depth and other structural changes, supporting the role of the neocortex in subcortical ischemic VaD 58. Lacunar infarcts and related microstructural alterations may also affect global brain volume or atrophy. To distinguish perivascular cavities or spaces, it has been suggested that lacunes be classed into three subtypes: lacunar infarcts, lacunar hemorrhages and dilated perivascular spaces 123. Lacunar infarcts usually result from progressive SVD manifested as hypertensive angiopathy or possibly microthrombi that may involve stenosis caused by hyalinosis. Apart from critical lesions occurring often in the internal capsule or caudate nucleus, there appear to be no pathological differences between symptomatic and asymptomatic patients. Perivascular edema and thickening, inflammation and disintegration of the arteriolar wall were common, whereas vessel occlusion was rare 4. Occasionally, lacunes may represent small hemorrhages or dilated perivascular spaces without infarction or hemorrhage. Microlacunes have also been described in SVD that essentially should be thought of as large cystic microinfarcts.

### WM changes

Diffuse and focal WM lesions are another prominent hallmark of SVD 54. Neuroimaging and pathological studies show that WM changes are invariably associated with cognitive abnormalities 23, 82, 90. WM alterations are much more profound in the hereditary SVDs such as CADASIL and CARASIL. They may occur in the absence of lacunar infarcts and extensive WM hyperintensities (WMH) appear associated with increased brain volume, particularly in CADASIL 125. In this disorder, WMH relate not only to loss of WM components but also to a global increase of water content in the cerebral tissue. WM hyperintensities on T2‐weighted MRI or leukoaraiosis as a decreased signal on CT may not only incorporate WM rarefaction, incomplete infarction, lacunar strokes, perivascular spacing and demyelination, but sometimes also axonal and Wallerian degeneration. Diffusion tensor imaging 89 has demonstrated how tissue microstructural changes in WM tracts and subcortical regions, for example, putamen and thalamus, are related to worsening clinical outcomes in SVD and CADASIL. The widespread WM axonal changes, particularly in the frontal lobe, may arise from differential stenosis and sclerosis of arterioles 20, possibly affecting certain axon bundles connecting to targets in the subcortical structures, specifically degeneration of thalamocortical pathways causing cortical atrophy 59.

There is some controversy as to whether deep or periventricular lesions are of greater importance but this depends on the definition of boundaries between the periventricular and deep WM 66. While lacunar infarcts are produced when the ischaemic damage is focal and of sufficient severity to result in a small area of necrosis, diffuse WM change is considered a form of rarefaction or incomplete infarction, where there may be selective damage to some cellular components. Although the U‐fibers are frequently spared, WM disease may comprise several patterns of alterations including pallor or swelling of myelin, loss of oligodendrocytes, axons and myelin fibers, cavitations with or without presence of macrophages and areas of reactive astrogliosis 100, where the astrocytic cytoplasm and cell processes may be visible with standard stains. Lesions in the WM also include spongiosis, that is, vacuolization of the WM structures and widening of the perivascular spaces 123.

Whereas WM changes focus on narrowing of the arterial system, in many cases, occlusion of veins and venules by collagenous thickening of the vessel walls also occur. The thickening of the walls of periventricular veins and venules by collagen (collagenosis) increases with age, and perivenous collagenosis (see previous discussion) increases in concert with leukoaraiosis 8. The presence of apoptotic cells in WM adjacent to areas of leukoaraiosis suggests that such lesions are dynamic, with progressive cell loss and expansion 7. Vascular stenosis caused by collagenosis may induce chronic ischemia or edema in the deep WM leading to capillary loss and more widespread effects on the brain 9, 10.

### Perivascular spaces (PVS)

Dilated PVSs are a frequent finding in the pathology of SVD (Figure 2). Both neuroimaging and pathological studies show that the severity of dilated PVS increases with age regardless of the brain region. Previous neuroimaging studies have indicated that the size of dilated PVS in the basal ganglia and frontal lobe WM correlates with cognitive impairment 21, 91. In CADASIL, the severity of dilated PVS in the temporal lobes or subinsular areas was found strongly and specifically related to the extent of WMH 126. Consistent with this finding, we previously demonstrated that increased volumes of PVS were related to WM myelin protein changes 123, suggesting that reduction in WM volume is one factor that creates PVS. Another important factor involved in the expansion of PVS is likely the lack of drainage of solutes including degraded proteins 15.

### Incomplete infarcts

Larger areas of incomplete infarction may extend into the WM 54. These are characterized by mild to moderate loss of oligodendrocytes, myelin and axons in areas where there may be hyalinized vessels 13. The parenchymal changes are normally accompanied by astrogliosis, some microgliosis and macrophage infiltration with usually no quantification of such response. The morphology of incomplete or subinfarctive changes, although suspected to be associated with cognitive function, is not consistently described in SVD. It may variably manifest as tissue rarefaction assessed by conventional stains and revealed as injury response such as microgliosis and astrocytosis, or the presence of other “reactive” cells or surrogate markers of dendritic, synaptic or axonal damage.

### Cerebral microinfarction

The accumulation of small, even miniscule, ischemic lesions is an important substrate of SVD 60. Microinfarcts may or may not involve a small vessel at its center but are foci with pallor, neuronal loss, axonal damage (WM) and gliosis. They are estimated to occur in their thousands and described as attenuated lesions of indistinct nature occurring in subcortical regions in sporadic SVD 24 and hereditary SVDs such as CADASIL 121. Microinfarction in the subcortical structures has been emphasized as a substrate of cognitive impairment 3, 60 and correlated with increased Alzheimer type of pathology, but cortical microinfarcts also appear to contribute to the progression of cognitive deficits in brain aging 67. Furthermore, microinfarcts even in border‐zone (watershed) regions may aggravate the degenerative process as indicated by worsening impairment in AD 106.

Neocortical microinfarcts are increased in the presence of CAA 84, 103, but are rarely observed in subcortical VaD linked to SVD 83, 106 or in CADASIL. However, cortical microinfarcts and, to lesser extent, periventricular demyelination were associated with cognitive decline in individuals at high risk for dementia 39. It is proposed that the changes in hemodynamics, for example, hypotension and atherosclerosis, may play a role in the genesis of microinfarcts in watershed regions.

### Cerebral microbleeds and hemosiderin

Cerebral microbleeds are an imaging construct to represent ferromagnetic hemosiderin iron derived from extravasation of erythrocytes. Cerebral microbleeds detected by T2‐W* or echo gradient MRI are also associated with histopathological evidence of lipohyalinosis and CAA 31. They are likely a surrogate marker of SVD evident on MRI along with lacunes and WM changes 111. The prevalence of radiological microbleeds in SVD ranges from 35% to 85%. Both radiological cerebral microbleeds and foci of hemosiderin containing single crystalloids, or larger perivascular aggregates, are found in the brains of older subjects including those diagnosed with VaD and CADASIL 72, but the radiological and pathological relationship between these findings has not been entirely clear. Recent evidence suggests that cerebral microbleeds detected by MRI are a surrogate marker for ischemic SVD rather than exclusively hemorrhagic diathesis 55. Greater putamen hemosiderin was significantly associated with indices of small vessel ischemia, including microinfarcts, arteriolosclerosis, perivascular spacing and lacunes in any brain region, but not large vessel disease or whole brain measures of neurodegenerative pathology. It appears that brain iron homeostasis and small vessel ischemic change are responsible for these rather than minor episodes of cerebrovascular extravasation.

## Assessment of Cerebrovascular Pathology

While there are agreed criteria for the assessment of various dementing disorders, including AD 27, 104, Parkinson disease dementia (PDD) 26, 29 and dementia with Lewy bodies 76, there are no widely accepted assessment criteria for VaD 56, 62. A plethora of literature exists summarizing the nature of cerebrovascular disease and associated pathologies, yet there are no standardized criteria for assessing and reporting cases, and therefore studies investigating vascular pathology rely on highly subjective routine or research‐based neuropathology reporting. For example, the National Institute of Aging‐Alzheimer's Association criteria recommended the assessment of hippocampal sclerosis, vascular brain injury and microvascular lesions in 12 regions, but did not specify how lesions should be quantified. Similarly, the BrainNet European consortium provided survey results from multiple centers investigating methods used for assessment of vascular pathology, comparing methods for brain processing and sampling, through to routine staining protocols, with the conclusion that there is much variation in the procedures 2. Further variability may come from lack of use of consistent terminology in reporting vascular pathologies 43. All these factors presumably lead to dichotomies in data with unclear comparisons between cohorts and datasets. We have attempted to develop cerebrovascular disease scoring tools in an attempt to bridge this gap, but no method has been widely accepted as the gold standard 24, 44, 62.

### Cerebral vessel sclerosis

The understanding of the cellular mechanisms on how cerebral arterial vessels alter with age or disease in the absence of atherosclerosis is still a matter for investigation. However, the degree of vessel wall changes is commonly reported in pathological assessments as a way of assessing extent of SVD (Figure 3). This tends to be qualitative and does not attempt to quantify the true burden. Nevertheless, some studies have attempted to quantify the pathological changes observed in several cerebrovascular diseases and provided insights as to how the extent of SVD burden varies in relation to hypertension, CADASIL and sporadic SVD (Table 2). The quantitative assessment of vascular wall changes provides a more sensitive method for comparing between diseases, which may appear qualitatively the same 19. Additionally, it could also be presumed that morphometric techniques for the purpose of measuring vessel wall thickness are less likely to encounter inter‐rater variations. Despite this, there still remains considerable variation in morphometrical methods used for assessing vessel wall stenosis, where different investigators use different section thickness, stains and equations to derive the final ratio of vessel wall to lumen changes (Table 2). The earliest methods on quantifying cerebral arterial thickening were derived by Furuyama 36 for assessing the effects of hypertension on systemic arteries. Furuyama's method required the radius of an artery's lumen to the midpoint of the medial layer “R” and the thickness of the medial layer “D” were used to calculate the degree of medial layer thickening. This equation was based on the assumption that the ratio between the radius, that is, distance from lumen center to central point of the media, and the thickness of medial layer, would increase as the medial layers become stretched and expanded with progression of disease, mostly due to uncontrolled hypertension. Okeda *et al* 87 utilized this method to assess medial layer thickening in the arachnoid and medullary arteries in several disorders including Binswanger's encephalopathy, CADASIL and hypertension. Despite some limitations of the study, for example, low sample numbers, their results provided clear evidence of the subtle differences in the extent of vessel wall changes between disorders, most notable that CADASIL vessel wall changes were more severe than those observed in Binswanger's disease 86. Prior to these studies, Lammie *et al* 70 developed the “sclerotic index” (SI) ratio, where the proportion of external and internal (luminal) diameter of the vessels is converted to a simple ratio to represent the extent of lumen narrowing. Using this system, three subclasses of SI were formulated, where a ratio of 0.2 to 0.3 denoted healthy vessels, a ratio of 0.3 to 0.5 was considered to be diseased and a ratio of over 0.5 was denoted as severe. Importantly, this study examined arterioles across the whole of the cerebrum and provided qualitative meaning to the SI ratio, using the three tier scale. They were also able to identify subtle differences to changes in vessels between the cortical and subcortical regions and compare the deep gray matter and WM. The SI ratio has since been widely used to estimate the extent of vessel wall degeneration and stenosis in morphometric studies. However, there is still no consensus on the most appropriate section thickness or size of vessels to be assessed. It is possible that the variations in the above parameters may explain the varying results. For example, while focusing on CADASIL, some studies using 5‐µm‐thick sections have reported mean SI to be in the region of 0.6–0.7 and for control cases as 0.4, whereas our studies using sections of 10 µm thickness indicate estimates comparable to Lammie *et al* 70: mean SI of 0.4 for CADASIL and 0.3 for controls.

**Table 2:** Morphological assessment of vessel wall thickening in SVD. Abbreviations: AD = Alzheimer's disease; CAA = cerebral amyloid angiopathy; CADASIL = cerebral autosomal arteriopathy with subcortical infarcts and leukoencephalopathy; H&E = hematoxylin and eosin; HERNS = hereditary endotheliopathy with retinopathy, nephropathy and stroke; hMID = hereditary multi‐infarct dementia MRI = magnetic resonance imaging; n/a = not applicable; NSD = no significant difference; PADMAL = pontine autosomal dominant microangiopathy and leukoencephalopathy; SVD = small vessel disease; VaD = vascular dementia WM = white matter

Quantitative pathology method	n subjects	Disease groups	Section thickness	Stain	Arteriole size	Brain regions	Typical SI findings	Reference
Ratio of wall thickness/total diameter	40 for MRI	Aged over 60 years	15 µm	Elastica van Gieson	Up to 150 µm	Frontal and occipital lobes	Normal WM; 0.17	Van Swieten 112
	19 for Neuropath						Severe WM damage; 0.29	
“Sclerotic rate” definition unclear	40	VaD n = 20	n/a	n/a	External diameter	Medullary arteries, basal ganglia	Sclerotic changes higher in VaD and AD compared with control	Furuta 35
		AD n = 20			<49 to >100 µm	Frontal lobe		
						Parietal lobe		
						Temporal lobe		
						Occipital lobe		
Sclerotic index (SI)	70	All autopsies	n/a	H&E	<approximately 300 µm diameter	Basal ganglia	“0.3 to 0.6”	Lammie *et al* 70
SI = 1‐(internal diameter/external diameter)		to laboratory				Thalamus	Severity of SVD evenly distributed within WM, basal ganglia and thalami	
						Frontal deep WM	SVD was slightly more severe in deep WM than deep gray matter	
						Parietal deep WM	In 9 cases pathology was more severe in deep gray matter	
						Temporal deep WM		
						Occipital deep WM		
“D” and D/R ratio	19	Binswangers encephalopathy (BE), n = 7	5 µm	Elastica‐Masson	Penetrating medullary artery	Frontal lobe	BE had greater frequency and extent of arterial intimal fibrosis	Tanoi *et al* 107
D is thickness medial layer, R is radius		Hypertensive brain hemorrhage (HH), n = 6			Depth of 12 000 µm		Some arteries showed complete segmental occlusion, with proximal lacunae	
		NT controls, n = 6			50 to over 200 µm		Complete occlusion was not seen in HH	
							NT cases had no intimal fibrosis or atheromas	
Measured vessel diameter	1	CADASIL, aged 75	n/a	Elastica‐Masson	>1000 µm	Frontal lobe	All medullary arteries had undergone medial wall thickening along complete length	Okeda *et al* 86
			probably 5 µm?		500–100 µm			
					100–500 µm			
					<100 µm			
D/R ratio	11	Malignant nephrosclerosis (HTN) (n = 5)	n/a	Elastica‐Masson	20–30 µm	Frontal lobe	D/R ratio was higher in MN (HTN) compared with NT controls	Okeda *et al* 85
		Normotensive controls (n = 6)	Probably 5 µm?		(end of medullary artery)			
D/R ratio	15	Binswanger's disease, n = 5	n/a	Elastica‐Masson	20–30 µm (end of medullary artery)	Frontal lobe	D/R values were increased in Binswanger's disease and hypertensive cases	Okeda *et al* 87
		Hypertensive controls, n = 5	Probably 5 µm?					
		Normotensive controls, n = 5			<100 µm and >100 µm			
SI = 1‐(internal diameter/external diameter)	13	CADASIL, n = 4	5 µm	H&E	External diameter	Frontal lobe	CADASIL; 0.75 in WM, 0.56 in gray matter	Miao *et al* 79
		Cerebrovascular controls n = 5			30–300 µm		CBV con; 0.41 in WM, 0.49 in gray matter	
		Non‐cerebrovascular controls n = 4					Non‐CBV con; 0.32 in WM, 0.47 in gray matter	
SI = 1‐(internal diameter/external diameter)	1 plus previous data	CADASIL aged 32	5 µm	H&E	External diameter 30–300 µm	Frontal lobe	CADASIL; 0.63 in WM, 0.55 in gray matter	Miao *et al* 77
		Control data from previous study						
SI = 1‐(internal diameter/external diameter)	17	CADASIL, n = 6 (including one young)	n/a	H&E	Internal diameter: <50 µm	Lenticular nucleus (caudo‐putamen)	Old CADASIL, 0.60; Young CADASIL, 0.57	Miao *et al* 78
		Old controls, n = 7					Old controls, 0.55; Young controls, 0.43	
		Young controls, n = 4				“Lobar controls”		
SI = 1‐(internal diameter/external diameter)	27	CAA, n = 10;	5 µm	H&E	External diameter: 30 to 300 µm	Frontal lobe	All frontal WM	Zhu *et al* 129
		VaD, n = 12;					Control; 0.38	
		Control, n = 5					VaD; 0.57	
							CAA; 0.53	
SI = 1‐(internal diameter/external diameter)	27	Young controls, n = 5	10 um	H&E	External diameter: 30–350 µm	Temporal pole	Young controls; 0.30	Yamamoto *et al* 123
		Old controls, n = 5					Old controls; 0.31	
		SVD, n = 8					SVD; 0.36	
		CADASIL, n = 9					CADASIL; 0.47	
SI = 1‐(internal diameter/external diameter)	30	Leukoaraiosis, n = 20	5 µm	H&E	Internal diameter <50 µm (<70 µm external diameter)	Frontal lobe	Both groups; 0.4 in gray matter	Huang *et al* 53
		Controls, n = 10					Control; 0.4 in WM	
		All elderly (60 to 78 yrs)					Leukoarioasis; 0.8 in WM	
SI = 1‐(internal diameter/external diameter)	50	Young controls, n = 11	10 µm	H&E	External diameter	Frontal lobe	Young controls; 0.29 in gray matter, 0.27 in WM	Craggs *et al* 19
		Old controls, n = 10			30–350 µm	basal ganglia	Old controls; 0.28 in gray matter, 0.26 in WM	
		SVD, n = 11					SVD; 0.30 in gray matter, 0.29 in WM	
		CADASIL, n = 9					CADASIL; 0.34 in gray matter, 0.38 in WM	
		PADMAL, n = 5					PADMAL; 0.30 in gray matter, 0.30 in WM	
		Swedish hMID, n = 4					Swedish hMID; 0.31 in gray matter, 0.32 in WM	
		HERNS, n = 1					HERNS; 0.34 in gray matter, 0.35 in WM	
SI = 1‐(internal diameter/external diameter)	21	Young controls, n = 10	10 µm	H&E	External diameter	Frontal lobe	All WM	Craggs *et al* 19
		CADASIL, n = 11			30–350 µm	Parietal lobe	Frontal; CADASIL, 0.45; young control, 0.32	
						Temporal lobe	Parietal; CADASIL, 0.4; young control, 0.3	
						Occipital lobe	Temporal; CADASIL, 0.43; young control, 0.3	
							Occipital lobe; CADASIL, 0.38; Young control, 0.27	
SI = 1‐(internal diameter/external diameter)	60	Aged controls, n = 10	10 µm	H&E	External diameter	Frontal lobe	Gray matter and WM	Foster *et al* 34
		Post‐stroke demented n = 10			35–350 µm		NSD between groups	
		Post‐stroke non‐demented n = 10					SI in WM 0.44 was higher than SI in GM (0.40)	
		AD, n = 10						
		Mixed, n = 10						
		VaD, n = 10						

Another important obvious factor to consider is the size of the vessels. Okeda *et al* 86 had first verified that the smaller the arteriole, the more severe the vascular smooth muscle loss. Similarly, Miao *et al* 79 showed that smaller arterioles exhibited more severe effects of vessel wall thickening on stenosis. In our study of several SVDs 19, we showed that smaller external diameters, followed by location were factors in differential involvement of arterioles between frontal lobe and basal ganglia locations. We also showed that there was an apparent low burden of severely sclerosed arterioles with a SI value of >0.5. Previous studies had reported SI values in CADASIL patients to be well over 0.5 in most cases; however, while we did observe some arterioles with SI considered severe, this was only 7.4% of vessels in the frontal lobe of CADASIL compared with 19.9% of vessels within the basal ganglia. In comparison, the older sporadic SVD subjects had only 6.3% arterioles in the severe category in the frontal lobe and 7.3% in the basal ganglia 19. The large difference between two diseases thought to mirror each other illustrates the power of morphometric assessment of arteriolar sclerosis and provides insights into potential mechanisms for differences in clinical presentations of subtypes of SVD. Our study also provided some potential for a threshold of SVD, which may be related to cognitive function. We found that the proportion of vessels within the healthy SI range was greater than 60% in cognitively normal controls, aged between 49 and 94 years of age, whereas in the SVD subjects, there were 36%–58% in the frontal lobe, and 35%–59% in the basal ganglia, indicating a subtle but potential difference in the cerebral tolerance to burden of vessel stenosis.

### WM changes

Another type of measurable cerebral change attributed to SVD is attenuation of the WM. We have previously shown that the greatest loss in myelin staining is encountered in VaD, most of which exhibited SVD 54. Furthermore, axonal abnormalities are also evident in SVD. It may be more useful to fully quantify the extent of WM damage rather than using a semiquantitative 0–3 scoring scale, as in our experience, a WM score of 0–1 is very rare in any cases aged over 40, and the scale of 2–3 does not differentiate between age‐associated changes in WM damage and extensive WM damage associated with dementia. We have previously reported on WM damage in young and old control cases without significant cognitive impairment and found that their WM damage may appear extensive enough to score 3 on the accepted scales 24, 102. While other reliable markers are urgently needed to assess both myelin and damaged axons, we showed that amyloid precursor protein immunoreactivity in axons correlates strongly with severe WM damage albeit in CADASIL 20. Quantitative measures for WM damage, either using histochemical stains or quantification of myelin staining using the myelin index, may allow further differentiation of subtle changes in WM pathology and delineate mechanisms in different disorders.

## Summary

The recognition of both sporadic and hereditary SVDs and their variants has enabled greater understanding of the heterogeneity of cerebrovascular disease. Recent advances in neuroimaging and quantitative vascular pathology demonstrate how SVD progresses and results in brain injury related to dementia. Quantification of degenerative changes within small cerebral vessels occurring during older age or caused by genetic defects are proposed to explain the progression of WM changes, resulting in leukoencephalopathy in both sporadic and monogenic SVDs. However, the initiating factors causing microvascular changes are the primary targets driving the progression of sporadic SVD need to be better understood.
